# Isotopic Differences between Forage Consumed by a Large Herbivore in Open, Closed, and Coastal Habitats: New Evidence from a Boreal Study System

**DOI:** 10.1371/journal.pone.0142781

**Published:** 2015-11-11

**Authors:** Marie-Andrée Giroux, Éliane Valiquette, Jean-Pierre Tremblay, Steeve D. Côté

**Affiliations:** 1 Département de Biologie and Centre d’Études Nordiques, NSERC Industrial Research Chair in Integrated Management of Resources of Anticosti Island, Université Laval, Québec, QC, Canada; 2 Center for Forest Research, Université Laval, Québec, QC, Canada; College of Charleston, UNITED STATES

## Abstract

Documenting habitat-related patterns in foraging behaviour at the individual level and over large temporal scales remains challenging for large herbivores. Stable isotope analysis could represent a valuable tool to quantify habitat-related foraging behaviour at the scale of individuals and over large temporal scales in forest dwelling large herbivores living in coastal environments, because the carbon (δ^13^C) or nitrogen (δ^15^N) isotopic signatures of forage can differ between open and closed habitats or between terrestrial and littoral forage, respectively. Here, we examined if we could detect isotopic differences between the different assemblages of forage taxa consumed by white-tailed deer that can be found in open, closed, supralittoral, and littoral habitats. We showed that δ^13^C of assemblages of forage taxa were 3.0‰ lower in closed than in open habitats, while δ^15^N were 2.0‰ and 7.4‰ higher in supralittoral and littoral habitats, respectively, than in terrestrial habitats. Stable isotope analysis may represent an additional technique for ecologists interested in quantifiying the consumption of terrestrial vs. marine autotrophs. Yet, given the relative isotopic proximity and the overlap between forage from open, closed, and supralittoral habitats, the next step would be to determine the potential to estimate their contribution to herbivore diet.

## Introduction

Determining habitat-related patterns in foraging behaviour is critical to better understand the ecology of large herbivores. For instance, the trade-off between using habitats rich in forage and those providing cover is generally recognized as a determinant of foraging behaviour in herbivores inhabiting forest ecosystems [1, 2]. Whereas forage is more abundant in open habitats due to direct exposure to sunlight [3, 4], vertical and lateral cover provide protection against extreme climatic conditions and predators, respectively [5–7]. Forage quality, however, may be higher under cover ([8], but see [9]) due to the delayed maturation of forage under shaded conditions [10]. In coastal areas, large herbivores may also use supralittoral and littoral habitats where they can forage on coastal plants [11], as well as on drifted seaweed ([12, 13], reviewed in [14]. Coastal plants can be fertilized by marine-derived nutrients [11, 15] and hence their quantity and quality may vary in parallel with the amount of drifted seaweed [11]. The biomass of seaweed available to terrestrial herbivores in littoral habitats is highly variable in space and time, as it depends on tidal movements, storms, coast orientation, and exposure to wind and waves [11, 16].

Sampling techniques have been developed to investigate the foraging behaviour of large herbivores (telemetry: e.g. [17], direct observations: e.g. [18]), yet quantifying the proportion of resources consumed in different habitats at the individual level and over large temporal scales remains challenging [19]. The stable isotope analysis (SIA) of animal tissues has proven to be an effective method to describe individual diet over small to large temporal scales [20]. SIA may represent an additional tool to quantify the habitat-related foraging behaviour of forest dwelling herbivores inhabiting coastal areas, both at the scale of individuals and over large temporal scales. Indeed, carbon (δ^13^C) and/or nitrogen (δ^15^N) isotopic signatures of forage can vary between open and closed forest habitats [21–24], between plants and seaweed [25–28], and between inland and coastal plants (e.g. [29]).

To quantify the proportion of different types of resources in a consumer’s diet using SIA, a statistical difference must be observed between the isotopic signatures of the different types of resources [30]. Hence, our first objective was to determine whether we could detect isotopic differences between open, closed, supralittoral, and littoral habitats using the isotopic signatures of carbon (δ^13^C) and nitrogen (δ^15^N) of forage taxa consumed by white-tailed deer (*Odocoileus virginianus;* hereafter *“deer”*). We predicted lower δ^13^C in closed than in open habitats [21–24], and lower δ^13^C and δ^15^N in terrestrial (open and closed) than in supralittoral [29] and littoral habitats [26–28]. Because different vegetation communities can occupy open and closed habitats, respectively, and isotopic signatures can vary between vegetation taxa, our second objective was to verify whether we could detect isotopic differences between open and closed forest habitats for a single species; we retained *Cornus canadensis* as the species of interest because this forage species was found in both open and closed habitats and is heavily used by deer in our study system [31, 32].

## Materials and Methods

### Ethics statement

To conduct field work on Anticosti Island, we obtained an authorisation for scientific studies from the Ministère des Ressources Naturelles et de la Faune du Québec (Permit number: 09052701409SF). The study area spans provincially-owned lands, and no protected species were sampled.

### Study area

**Fig 1:**
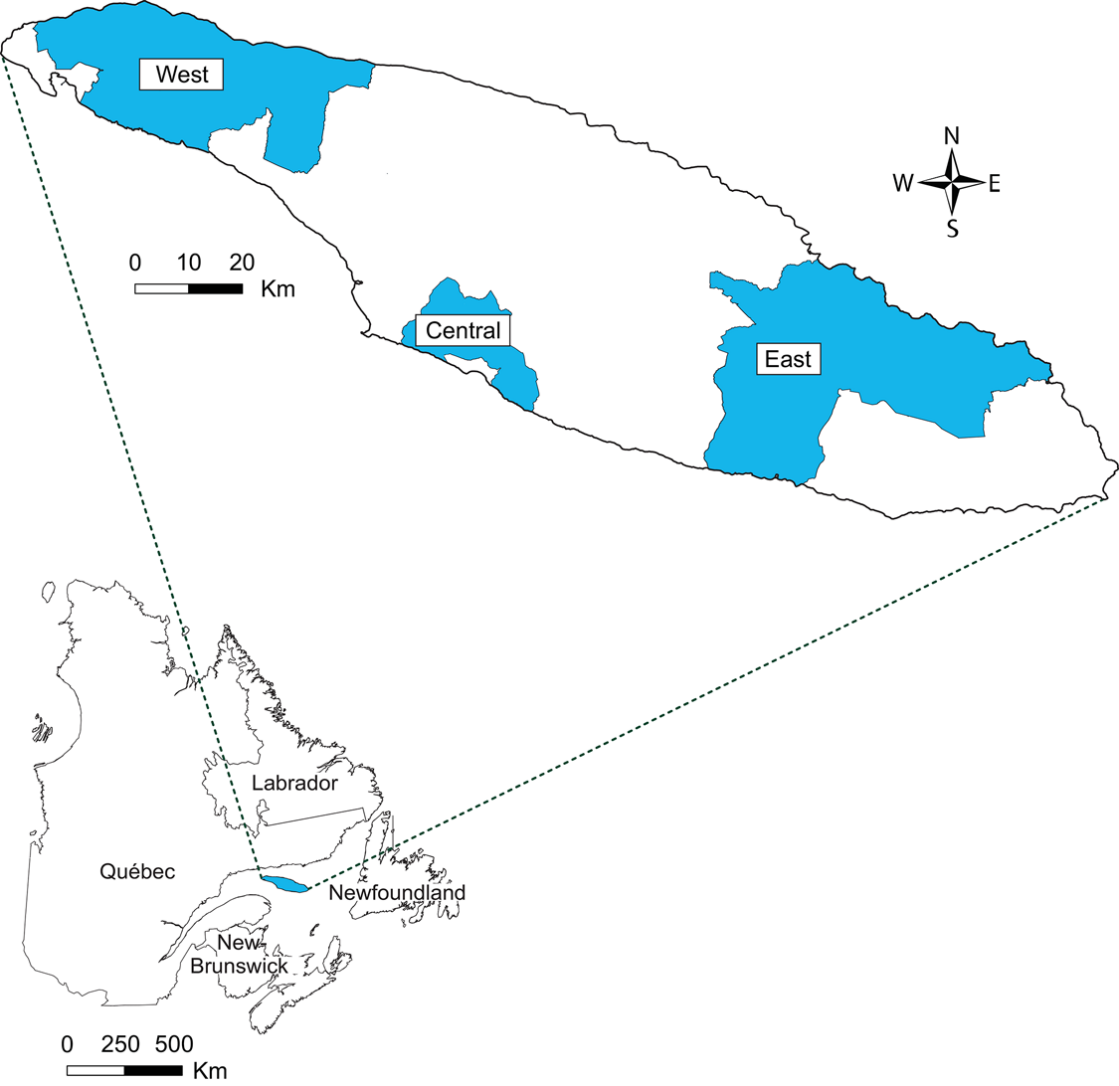
Anticosti Island (Canada) with the three sectors of the study area.

We conducted the study from 2002 to 2010 on Anticosti Island in the Gulf of St. Lawrence, Québec, Canada (49.06–49.95°N, 61.67–64.52°W; 7943 km^2^; Fig 1). The climate of Anticosti Island is maritime and characterized by cool summers and long winters. The mean monthly air temperature is 16°C in July and -11°C in January [33]. Snow precipitation averages 328 cm annually, while rainfall averages 61 cm [34]. Anticosti Island is dominated by closed coniferous forests and open habitats covering 56% and 36% of the island, respectively [35]. The forests of Anticosti Island belong to the boreal zone and are part of the eastern balsam fir-white birch bioclimatic region [36]. As a result of the introduction of approximately 220 white-tailed deer on this predator-free island at the end of the 19^th^ century, long term severe browsing induced the decline of dominant deciduous species such as white birch (*Betula papyrifera*) and trembling aspen (*Populus tremuloides*; [37]). Presently, Anticosti forests are dominated by balsam fir (*Abies balsamea*), white spruce (*Picea glauca*), and black spruce (*P*. *mariana*). The following open habitats are found on Anticosti Island: peatlands, canopy openings in forest stands (edaphic gaps or openings resulting from natural disturbances such as insect outbreaks or windthrowns events), clear-cuts resulting from commercial logging, and burned areas of natural origin. A supralittoral habitat dominated by graminoids and forbs is found at the periphery of the island at the ecotone between forest stands and beaches. Behavioural observations have shown that deer feed on plants in this habitat, as well as on washed-up seaweed in the adjacent littoral habitat (Giroux, Côté and Tremblay, *unpublished data*).

### Vegetation and seaweed sampling

We sampled vegetation and seaweed in the western sector of the island in July 2008, and we collected additionnal samples of *Cornus canadensis* in the central and eastern sectors of the island (Fig 1). In the western sector, we randomly selected 6 sites in closed coniferous forest, as well as in the supralittoral and the littoral habitats, and 3 sites in each of the following open habitats: fens, canopy openings, and clear-cuts. We randomly selected site locations using a forest map (1:20 000) that was generated by delineating habitat polygons using 1:15,000 aerial photographs taken in 1997, to which we added recent clear-cuts. Each site was defined as a 50-m radius area around the center of the location. At each site, except in littoral habitat, we aimed to collect at least one tablespoon of leaves (l), needles (n) or whole above-ground specimens (w) of the vegetation taxa consumed by deer on Anticosti Island during the summer, as assessed by micro-histological analyses of 15 pellet groups collected on Anticosti Island in July 2004 (list of taxa in S1 Table). We also aimed to collect two additional taxa known to contribute to deer diet in specific habitats, namely *Elymus arenarius* (supralittoral habitat; behavioral observations) and *Myrica gale* (fen; evidence of deer browsing on this taxum [38]). At each site, we thoroughly searched the 50-m radius area for all taxa in S1 Table, sampling those that were encountered and omitting those that were not found. Because some taxa were also excluded *a posteriori* when they were found in only one site per habitat (*n* = 1 per species in those cases), we ended up with the following list of taxa (hereafter “forage taxa”): *Abies balsamea* (n), *Carex spp*. (l), *Cornus canadensis* (l), *Dasiphora fruticosa* (l), *Elymus arenarius* (l), Ferns (l), *Fragaria* spp. (l), *Graminae* spp. (l), *Hieracium* spp. (l), *Juncaceae* spp. (l), Lichens (w), Mosses (w), *Myrica gale* (l), and *Vicia cracca* (l). The contribution of these taxa (excluding *E*. *arenarius* and *M*. *gale*) to summer diet of white-tailed deer on Anticosti Island summed up to 75% (S1 Table). We collected only the leaves or needles of most taxa because they represent 80% of deer summer and fall diets on Anticosti Island [39]. In littoral sites, we aimed to collect at least one tablespoon of thallus of the following seaweed taxa consumed by deer on Anticosti Island, as revealed by behavioural observations: *Laminaria* spp., *Fucus* spp., and *Ulva* spp. To collect enough *C*. *canadensis* to compare its isotope values between open and closed habitats, we also randomly sampled the following number of additional sites in each of the central and eastern sectors of the island: 6 sites in closed canopy, 3 sites in fens and canopy openings, as well as 3 sites in burned areas (eastern sector only). We preserved all samples in polyethylene airtight bags, froze them within 8 hours of collection, then processed and analyzed their δ^13^C and δ^15^N according to laboratory procedures described in S1 Methods.

### Statistical analyses

#### Objective 1

We compared isotopic signatures of vegetation and seaweed samples between the 6 following habitat categories: closed, fens, canopy openings, clear-cuts, supralittoral, and littoral. We used univariate linear mixed models (LMMs) to quantify the influence of habitat on δ^13^C and δ^15^N on deer forage, respectively. We specified the site as a random variable. Because we did not find all consumed taxa in every site of the same habitat, we also specified taxa as a random variable to take into account that sample sizes differed between taxa within an habitat (Table 1). We used multiple comparisons to compare the differences in isotopic signatures between all open habitats (fens, canopy openings, and clear-cuts althogether) and closed, supralittoral, or littoral habitats, as well as between all terrestrial habitats (open and closed habitats) and supralittoral or littoral habitats. Because the assumption of homogeneity of residuals was not respected for δ^15^N, we modelled the variance structure to allow residual variance to vary between habitats [40].

**Table 1:** Number of samples per taxa and habitat used to estimate the isotopic differences between habitats on Anticosti Island, Québec (Canada).

TAXA	HABITAT					
	Closed canopy	Open			Supralittoral	Littoral
		Canopy opening	Cut	Peatland		
***Abies balsamea***	3	3				
***Carex* spp.**	6	3		3		
***Cornus canadensis***	6	3	3			
***Elymus arenarius***					6	
**Ferns**	6	2	3			
***Fragaria* spp.**	3					
**Graminae spp.**	6	3	3		6	
***Hieracium* spp.**	3	2	2		3	
***Juncaceae* spp.**				3	5	
**Lichens**	4	3				
**Mosses**	5	2	3	3		
***Myrica gale***				3		
***Dasiphora fruticosa***				3		
***Vicia cracca***					3	
***Laminaria* spp.**						6
***Fucus* spp.**						6
***Ulva* spp.**						6
**TOTAL**	**42**	**21**	**14**	**15**	**23**	**18**

#### Objective 2

To verify whether we could detect isotopic differences between open and closed habitats in a single preferred taxum (*C*. *canadensis*), we fitted linear models to quantify variations in δ^13^C and δ^15^N, respectively, between open and closed habitats. We retained *C*. *canadensis* because it was the only forage species sampled in sufficient number in both open and closed habitats. We also took into account the sector and its interaction with habitat in the set of models compared to verify whether the eventual difference between habitats was maintained across sectors.

For both objectives, we used Akaike’s Information Criterion corrected for small sample size (AICc; [41]) to select the best-fitting model. Unless otherwise mentionned, all analyses respected the assumptions of normality and homoscedasticity of residuals and were performed with R version 2.15.1 [42].

## Results

### Objective 1: Isotopic differences between habitats

**Fig 2:**
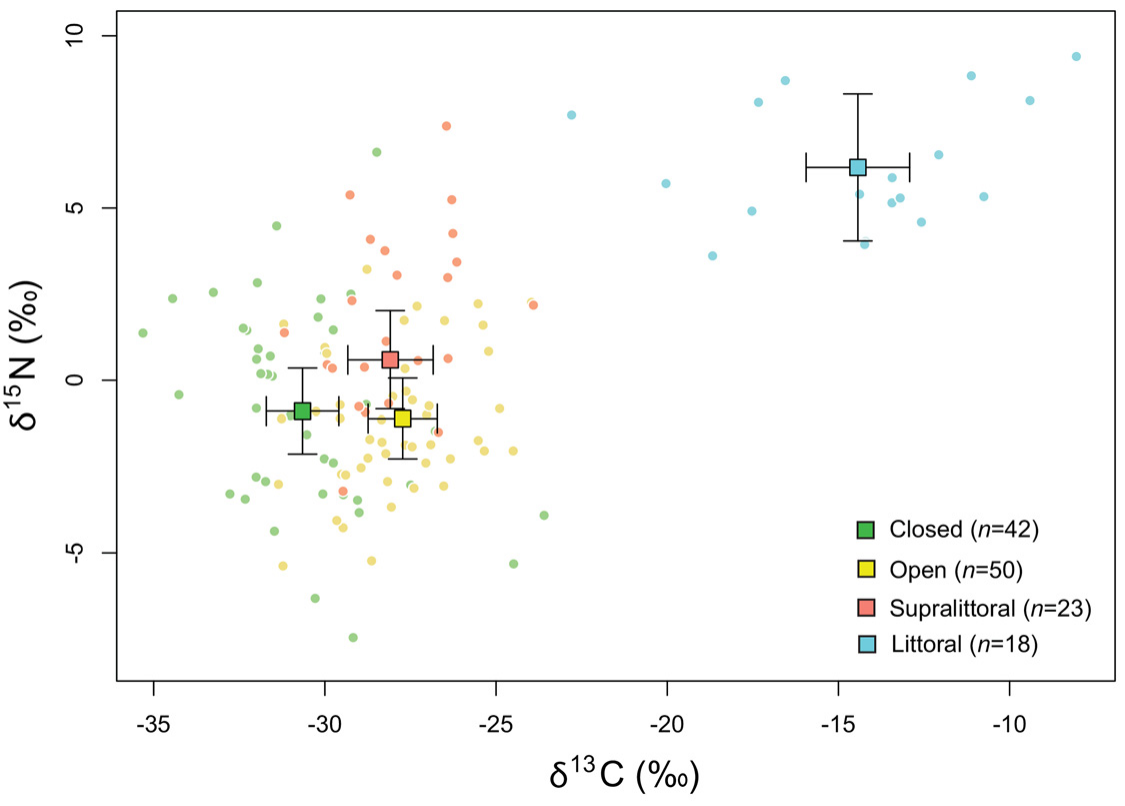
Carbon and nitrogen isotopic signatures (δ^13^C and δ^15^N) of habitat-specific assemblages of forage taxa used by white-tailed deer on Anticosti Island, Canada. We show the average signatures (± SD) of the four habitat types.

δ^13^C of forage taxa was 3.0‰ (95% CI = [1.3, 4.6]), 2.6‰ (95% CI = [1.0, 4.1]), and 16.2‰ (95% CI = [14.4, 18.1]) higher in open, supralittoral, and littoral habitats, respectively, than in the closed habitat (Fig 2). δ^15^N did not differ between closed (-0.9‰, 95% CI = [-2.1, 0.4]) and open (mean = -1.1‰, 95% CI = [-2.3, 0.1]) habitats, but was 2.0‰ (95% CI = [0.3, 3.7]) and 7.4‰ (95% CI = [4.5, 10.1]) higher in supralittoral and littoral habitats, respectively, than in terrestrial habitats (closed and open habitats; Fig 2).

The isotopic difference in δ^13^C tended to be larger between the closed habitat and peatlands (4.4‰, 95% CI = [2.6, 6.1]), than between the closed habitat and the canopy openings (1.9‰, 95% CI = [0.3, 3.5]) or cuts (2.5‰, 95% CI = [0.8, 4.2]). δ^15^N did not differ between the closed habitat and any of the open habitats (peatlands: 0.4‰, 95% CI = [-1.3, 2.1], canopy openings: 0.2‰, 95% CI = [-1.3, 1.7], cuts: -1.4‰, 95% CI = [-2.9, 0.2]).

### Objective 2: Isotopic differences in a single species between open and closed habitats

For *Cornus canadensis*, the best model explaining variation in isotopic signatures included only habitat as a predictor variable for δ^13^C and included no variables for δ^15^N (null model; see S2 Table). δ^13^C was 2.6 ‰ (95% CI = [1.7, 3.5] higher in open (mean = -27.7‰, 95% CI = [-28.3, -27.0], *n* = 15) than in closed (mean = -30.3‰, 95% CI = [-30.9, -29.6], *n* = 14) habitats. δ^15^N did not differ between open (mean = -1.8‰, 95% CI = [-2.9, -0.8]) and closed (mean = -2.3‰, 95% CI = [-3.4, -1.2]) habitats.

## Discussion

We showed an isotopic distinction between forage consumed by a large herbivore in open, closed, supralittoral, and littoral habitats. As predicted, δ^13^C of forage consumed by deer was lower in closed than in open, supralittoral, and littoral habitats, whereas δ^15^N was lower in terrestrial (open and closed) than in supralittoral and littoral habitats. The difference between δ^13^C of forage found in the closed habitat and in the different open habitats ranged from 1.9‰ to 4.4‰. When comparing isotopic signatures between open and closed habitats for a single preferred plant species, *C*. *canadensis*, we also observed higher δ^13^C in closed than in open habitats.

### Isotopic differences between habitats

Detecting variations in the isotopic signatures of deer forage taxa between habitats was the first step in evaluating the potential for using SIA to quantify the contribution of resources from the mosaic of habitats found in coastal boreal forests to deer diet. The isotopic difference between open and closed habitats observed in our study (3.0‰ on average) was slightly larger than in other studies conducted in boreal forests (0–2.2‰: [22], reviewed in [23] and [43]), and this holds for cuts (2.5‰) and peatlands (4.4‰), but not for canopy openings (1.9‰). The isotopic differences between terrestrial vegetation (open and closed habitats) and seaweeds (littoral habitats) were similar to the ranges reported in the literature (our study: δ^13^C = 14.8‰ and δ^15^N = 5.9‰; other studies: δ^13^C = [9.3‰,19‰] and δ^15^N = [6.4‰,9‰], [26–28]). The difference in δ^15^N between inland vegetation and coastal plants was 2.6‰, which is smaller than what has been observed for inland and coastal terrestrial plants in Africa (5–10‰; [29]), but closer to what has been observed for inland and coastal terrestrial plants in northern California (~3‰;[29]).

Our ability to explain the differences or similarities in the extent of isotopic contrasts compared to previous studies is limited by the fact that the mechanisms underlying isotopic differences between autotrophs are still largely hypothetical. To explain the isotopic differences between open and closed habitats, two potential mechanisms have been identified, but both remain to be tested. These mechanisms are: 1) the lesser extent of mixing of the ground level air layer (where ^13^C-depleted CO_2_ derived from litter decomposition is released) with the overlying air in less ventilated closed habitats [44], and 2) the influence of light level on the isotopic fractionation of carbon during photosynthesis [45]. The isotopic contrast between δ^13^C in terrestrial and marine autotrophs may result from the use of bicarbonate, which has a higher δ^13^C than dissolved CO_2_, as a source of carbon by algae and/or from the slower diffusion of CO_2_ in water that can influence isotopic fractionation of carbon [46]. In marine ecosystems, the presence of ^15^N-containing compounds that are enriched in ^15^N compared to sources of nitrogen in terrestrial ecosystems can likely explain the higher δ^15^N observed in marine autotrophs [47]. Coastal plants may have access to ^15^N-enriched nitrate from sea-spray, potentially explaining their higher δ^15^N compared to terrestrial plants [48]. Although our study was not designed to study the mechanisms underlying the isotopic contrast between open and closed habitats discussed above, we nonetheless showed that such a contrast could be observed in a single species sampled in both open and closed habitats (*C*. *canadensis*). The isotopic difference between *C*. *canadensis* sampled in open and closed habitats (2.6‰) was within the range of the differences observed between open and closed habitats when comparing multiple taxa consumed by deer (1.9‰ to 4.4‰). This result indicates that the isotopic difference between open and closed habitats is not simply due to different vegetation communities occupying the two habitat types (Table 1). Indeed, isotopic signatures can vary between vegetation taxa due to differences in anatomy, physiology, and biochemistry [49]. For example, conifers are enriched in ^13^C compared to deciduous trees because of different photosynthesis/transpiration ratios [50]. Our study showed that a canopy effect can occur without variations associated with habitat-specific vegetation assemblages.

Further studies should aim at clearly assessing whether isotopic differences among habitats is a consequence of intraspecific variation in isotopic ratios among habitats, differences in community composition or both. In our study system, this could be performed by working at the species level rather than at the genera or higher taxonomic rank. Performing the study at the species level would likely result in both a larger set of species unique to each habitat and a larger set of species that could be sampled in more than one habitat (e.g. *C*. *canadensis* in our study). This would allow to compare the isotopic differences among habitats obtained when analysing all species, species unique to certain habitats only, and species found in all habitats (or in pairs of habitats). In our study, working at the species level was limited by the resolution of micro-histological analyses of deer diet (S1 Table). In the future, this limit could be relaxed by conducting diet analyses using DNA barcoding.

### Using habitat-specific isotopic signatures to reconstruct the diet of coastal herbivores

In our study, the isotopic values of forage from open, closed and supralittoral habitats were relatively similar compared to the isotopic values frequently used in diet reconstruction studies (e.g. the contrasting isotopic values of marine and terrestrial autotrophs; [25, 26, 28]). In addition, there was a high degree of overlap between isotopic values from closed, open and supralittoral habitats. Given the relative isotopic similarity and the overlap between forage from open, closed, and supralittoral habitats, the next step would be to determine whether using these resources in isotopic diet reconstruction may help understand the relationships between diet estimates and predictors describing environmental or individual variations. Recent advances in isotopic diet reconstruction have stressed the need to account for different sources of uncertainty in isotopic analyses, even though doing this would result in uncertainty around diet estimates [51, 52]. All other variables being equal, smaller isotopic contrasts induce greater uncertainty around isotopically derived diet estimates [52, 53]. Although diet estimates obtained with such models are more robust, both interpretation and statistical analyses should take this uncertainty into account [52, 54]. Hence, further studies should determine whether the estimated contributions of forage from open, closed, and supralittoral habitats to the diet of herbivores would be precise enough to either show the effects of predictors describing environmental or individual variations on those diet estimates or an effect of diet variation on individual performance.

## Conclusions

Determining how the use of open, closed, and coastal habitats influences the performance of forest dwelling large herbivores would deepen our understanding of how they adjust their foraging behaviour to maximize survival, growth and reproduction when exposed to various trade-offs (e.g. forage quantity vs. quality or forage quantity vs. predation risk). SIA may represent an additional technique to address such questions, and may prove especially useful to determine the influence of consumption of terrestrial vs. marine autotrophs on the performance of herbivores. Yet, the potential to address such questions with respect to the relative use of forage from open, closed, and supralittoral habitats remains to be demonstrated.

## Supporting Information

S1 Data Data set used in objective 1 to verify whether we could detect isotopic differences among habitats. (XLSX)

S2 Data Data set used in objective 2 to verify whether we could detect isotopic differences between open and closed habitats in a single preferred taxum (*C*. *canadensis*). (XLSX)

S1 Methods Additional methodological details of isotopic analyses. (DOCX)

S1 Table Contribution of different vegetation taxa to the summer diet of white-tailed deer on Anticosti Island during summer, as assessed by micro-histological analyses of 15 pellet groups collected on Anticosti Island (Québec, Canada) in July 2004 (data from Massé and Côté). Taxa in bold were those included in the comparison of isotopic signatures between habitats described in the main text.
(DOCX)

S2 Table Model selection for linear models fitted to determine if δ^13^C (A) and δ^15^N (B) of *Cornus canadensis* varied between habitat (open terrestrial vs. closed), sector (western, central, and eastern) and their interaction. We report the number of parameters (*k*), Akaike’s information criterion for small sample sizes (AICc) relative to the model with the lowest AICc (ΔAICc), as well as the AICc weight (ωAICc). Models are ranked by their AICc values. The best model is shown in bold.
(DOCX)
