# Supplementary material for: Isotopic Differences between Forage Consumed by a Large Herbivore in Open, Closed, and Coastal Habitats: New Evidence from a Boreal Study System
Source: PLoS One. 2015 Nov 11;10(11):e0142781. doi: 10.1371/journal.pone.0142781 (PMC4641657; doi:10.1371/journal.pone.0142781)
Supplement: S1 Methods — (DOCX) [file pone.0142781.s003.docx]

**S1 Methods: Additional methodological details of isotopic analyses**

## Isotopic Differences Between Forage Consumed By A Large Herbivore In Open, Closed, And Coastal Habitats: New Evidence From A Boreal Study System

Marie-Andrée Giroux^1^*^,#a^, Éliane Valiquette^1^, Jean-Pierre Tremblay^1,2^ and Steeve D. Côté^1^*

^1^ Département de Biologie and Centre d’Études Nordiques, NSERC Industrial Research Chair in Integrated Management of Resources of Anticosti Island, Université Laval, Québec, Qc, Canada

^2^ Center for Forest Research, Université Laval, Québec, Qc, Canada

^#a^ Present address: Département de biologie, Canada Research Chair in Polar and Boreal Ecology, Université de Moncton, Moncton, Nb, Canada

*Corresponding authors.

E-mail: [marie.a.giroux@gmail.com](mailto:marie.a.giroux@gmail.com); [steeve.cote@bio.ulaval.ca](mailto:steeve.cote@bio.ulaval.ca)

**Processing samples**

Prior to analyses, we removed dead tissues of forage samples, cleaned tissues (leaves, needles, complete above-ground specimens, and thallus) with distilled water and then with ethanol 95%, and dried them under a fume-hood. We oven-dried these samples at 60^o^C for 48 hours. We ground forage samples in a fine powder using a ballmill grinder, and loaded approximately 1 mg of forage samples in tin capsules. We combusted tin capsules in a Carlo Erba NC2500 elemental analyzer. Resultant gases (CO_2_ and N_2_) were delivered via continuous flow to a Finnigan Mat Delta Plus isotope ratio mass spectrometer and analyzed for stable isotopes of carbon and nitrogen. Stable isotope ratios are expressed in δ-values as parts per thousand (‰) deviations from standards, namely Pee Dee Belemnite for C and atmospheric air for N, following δ^13^C = [(^13^C/^12^C sample-^13^C/^12^C standard)/ ^13^C/^12^C standard] X 1000 and δ^15^N = [(^15^N/^14^N sample-^15^N/^14^N standard)/ ^15^N/^14^N standard] X 1000. Analytical error is reported by providing measures of accuracy and precision ([Jardine and Cunjak 2005](#_ENREF_2)). We estimated accuracy with measurements (mean ± SD) of a commercially available standard (acetanilide, Elemental Microanalysis Ltd.: δ^13^C = -28.5 ± 1.6‰ SD and δ^15^N = -2.1 ± 0.2‰ SD, *n* = 94). Precision was measured across runs using peach leaf (SD: δ^13^C = ± 0.1‰ and δ^15^N = ± 0.2‰, *n* = 20). We also measured precision within analytical runs using duplicate samples. Average absolute difference between duplicates was 0.1 ± 0.1‰ SD and 0.2 ± 0.2‰ SD (*n* = 39) for δ^13^C and δ^15^N, respectively.

**References**

Jardine TD, Cunjak RA (2005) Analytical error in stable isotope ecology. Oecologia 144:528-533
