# Supplementary material for: Isotopic Differences between Forage Consumed by a Large Herbivore in Open, Closed, and Coastal Habitats: New Evidence from a Boreal Study System
Source: PLoS One. 2015 Nov 11;10(11):e0142781. doi: 10.1371/journal.pone.0142781 (PMC4641657; doi:10.1371/journal.pone.0142781)
Supplement: S1 Table — Taxa in bold were those included in the comparison of isotopic signatures between habitats described in the main text. (DOCX) [file pone.0142781.s004.docx]

**S1 Table. Contribution of different vegetation taxa to the summer diet of white-tailed deer on Anticosti Island, as assessed by micro-histological analyses of 15 pellet groups collected on Anticosti Island in July 2004 (data from Massé and Côté).** Taxa in bold were those included in the comparison of isotopic signatures between habitats described in the main text.

| TAXA | % IN SUMMER DIET |
| --- | --- |
| ***Cornus canadensis*** | **17.7** |
| ***Hieracium* spp.** | **12.3** |
| ***Abies balsamea*** | **9.3** |
| **Grasses** | **9.1** |
| **Ferns** | **6.2** |
| **Mosses** | **5.4** |
| *Achillea millefolium* | 4.3 |
| ***Fragaria* spp.** | **4.2** |
| *Equisetum* spp. | 4 |
| ***Dasiphora fruticosa*** | **3.5** |
| *Galium* spp. | 3.4 |
| **Lichens** | **3.2** |
| ***Vicia cracca*** | **2** |
| *Comandra* spp. | 1 |
| Other forbs | 1 |
| *Trifolium* spp. | 1.4 |
| *Rubus* spp. | 1.2 |
| *Geum macrophyllum* | 1.1 |
| *Eriophorum* spp. | 1.1 |
| ***Juncus* spp.** | **1.1** |
| *Dactylis glomerata* | 0.9 |
| *Senecio* spp. | 0.8 |
| *Picea* spp. | 0.8 |
| ***Carex* spp.** | **0.7** |
| *Gaultheria hispidula* | 0.7 |
| *Coptis groenlandica* | 0.6 |
| Other shrub leaf | 0.6 |
| *Lycopodium* spp. | 0.6 |
| *Anaphalis margaritacea* | 0.6 |
| *Aster* spp. | 0.5 |
| *Taraxacum officinale* | 0.5 |
| *Vaccinium* spp. | 0.3 |
| *Pyrola* spp. | 0.3 |
