# Supplementary material for: Isotopic Differences between Forage Consumed by a Large Herbivore in Open, Closed, and Coastal Habitats: New Evidence from a Boreal Study System
Source: PLoS One. 2015 Nov 11;10(11):e0142781. doi: 10.1371/journal.pone.0142781 (PMC4641657; doi:10.1371/journal.pone.0142781)
Supplement: S2 Table — We report the number of parameters (k), Akaike’s information criterion for small sample sizes (AICc) relative to the model with the lowest AICc (ΔAICc), as well as the AICc weight (ωAICc). Models are ranked by their AICc values. The best model is shown in bold. (DOCX) [file pone.0142781.s005.docx]

**S2 Table.** **Model selection for linear models fitted to determine whether δ^13^C (A) and δ^15^N (B) of *Cornus canadensis* varied between habitats (open terrestrial vs. closed), sectors (western, central, and eastern) and their interaction.** We report the number of parameters (*k*), Akaike’s information criterion for small sample sizes (AICc) relative to the model with the lowest AICc (ΔAICc), as well as the AICc weight (ωAICc). Models are ranked by their AICc values. The best model is shown in bold.

A- δ^13^C (*n*=29)

| Model | *k* | AICc | ΔAICc | ωAICc |
| --- | --- | --- | --- | --- |
| **Habitat** | **3** | **98.1** | **0.0** | **0.80** |
| Habitat + Sector | 5 | 101.3 | 3.2 | 0.16 |
| Habitat x Sector | 7 | 104.0 | 5.9 | 0.04 |
| Null model | 2 | 119.1 | 21.0 | 0.00 |
| Sector | 4 | 123.1 | 25.0 | 0.00 |

A- δ^15^N (*n*=29)

| Model | *k* | AICc | ΔAICc | ωAICc |
| --- | --- | --- | --- | --- |
| **Null model** | **2** | **126.3** | **0.0** | **0.68** |
| Habitat | 3 | 128.4 | 2.1 | 0.24 |
| Sector | 4 | 131.0 | 4.7 | 0.06 |
| Habitat + Sector | 5 | 133.5 | 7.2 | 0.02 |
| Habitat x Sector | 7 | 139.5 | 13.2 | 0.00 |
